# Interplay between CO_2_ and light governs carbon partitioning in *Chlamydomonas reinhardtii*

**DOI:** 10.1111/ppl.14630

**Published:** 2024-11-19

**Authors:** Luca Zuliani, Michela Cecchin, Tea Miotti, Matteo Paloschi, Stephan Cuine, Stefano Cazzaniga, Yonghua Li‐Beisson, Matteo Ballottari

**Affiliations:** ^1^ Dipartimento di Biotecnologie Università di Verona Verona Italy; ^2^ Aix‐Marseille Univ, CEA, CNRS Institute of Biosciences and Biotechnologies of Aix‐Marseille Saint‐Paul‐lez Durance France

## Abstract

Increasing CO_2_ availability is a common practice at the industrial level to trigger biomass productivity in microalgae cultures. Still, the consequences of high CO_2_ availability in microalgal cells exposed to relatively high light require further investigation. Here, the photosynthetic, physiologic, and metabolic responses of the green microalga model *Chlamydomonas reinhardtii* were investigated in high or low CO_2_ availability conditions: high CO_2_ enabled higher biomass yields only if sufficient light energy was provided. Moreover, cells grown in high light and high CO_2_ availability were characterized, compared to cells grown in high light and low CO_2_, by a relative increase of the energy‐dense triacylglycerols and decreased starch accumulation per dry weight. The photosynthetic machinery adapted to the increased carbon availability, modulating Photosystem II light‐harvesting efficiency and increasing Photosystem I photochemical activity, which shifted from being acceptor side to donor side limited: cells grown at high CO_2_ availability were characterized by increased photosynthetic linear electron flow and by the onset of a balance between NAD(P)H oxidation and NAD(P)^+^ reduction. Mitochondrial respiration was also influenced by the conditions herein applied, with reduced respiration through the cytochrome pathway compensated by increased respiration through alternative pathways, demonstrating a different use of the cellular reducing power based on carbon availability. The results suggest that at high CO_2_ availability and high irradiance, the reducing power generated by the oxidative metabolism of photosynthates is either dissipated through alternative oxidative pathways in the mitochondria or translocated back to the chloroplasts to support carbon assimilation and energy‐rich lipids accumulation.

## 1 INTRODUCTION

The continuous rise of the atmospheric CO_2_ level is leading to a severe global warming environmental crisis. Microalgae‐based CO_2_ sequestration is a promising platform for sequestering inorganic carbon back into the biosphere, reducing greenhouse effects, and developing sustainable bio‐based industrial processes (Vecchi et al., 2020). Understanding the process underlying high CO_2_ growth is crucial to this process. Microalgae fix CO_2_ through their photosynthetic process. Harvesting and conversion of light energy into chemical energy occur in the thylakoidal membranes in the chloroplast by the photochemical activity of pigment‐binding protein complexes called Photosystem I (PSI) (Mazor et al., 2017) and II (PSII) (Shen et al., 2019), which in oxygenic photosynthetic organisms, as eukaryotic microalgae, work in series to strip electrons from water and transfer them to NADP^+^ producing NADPH and generating an electrochemical gradient used by ATPase to synthesize ATP. These complexes comprise a core complex, where photochemical reactions occur, and an external antenna system, increasing light‐harvesting efficiency (Gao et al., 2018; Pan et al., 2020). The Calvin Benson cycle requires both ATP and NADPH as the source of chemical energy and reducing power, respectively, to fix CO_2_ into sugars. While both ATP and NADPH are produced by linear electron flow (LEF), only ATP is produced by cyclic electron flow (CEF), where electrons driven by PSI activity are recycled to plastoquinone (Burlacot et al., 2022; Alric, 2014).

In parallel, photosynthates produced by the chloroplast are used either as building blocks for biosynthesis or are transported into the cytosol, potentially entering an oxidative pathway that produces reducing power as NADH and, in mitochondria, ATP by oxidative phosphorylation (Cecchin et al., 2021; Burlacot et al., 2019; Uhmeyer et al., 2017). A constant balance between chloroplast and mitochondrial activity is fundamental for cell survival and adaptation to fluctuating environmental conditions (Uhmeyer et al., 2017). Considering the low diffusion rate of CO_2_ in a water environment, several microalgae species evolved a system to enrich the CO_2_ concentration inside the cell, called the carbon concentrating mechanism (CCM) (Gee and Niyogi, 2017; Raven and Beardall, 2016; Wang et al., 2015; de Araujo et al., 2011; Yamano et al., 2008). Upon CCM activation, inorganic carbon is actively transported in the cell and in particular in a specific compartment in the chloroplast called pyrenoid, where carbonic anhydrase and the enzyme responsible for CO_2_ fixation, i.e., the Ribulose‐1,5‐bisphosphate carboxylase/oxygenase (RuBisCO) enzyme, are located (Wang et al., 2015). Low CO_2_ concentrations induce the CCM mechanism (Wang et al., 2015) mainly through the transcription regulator CIA5 (Moroney et al., 1989; Fukuzawa et al., 2001; Xiang et al., 2001). A recent report demonstrates that CCM requires energy provided by CEF and flavodiiron protein‐dependent oxygen photoreduction (Burlacot et al., 2022).

CO_2_ availability was also reported to influence the induction of photoprotective mechanisms in different microalgal species. In the case of *C. reinhardtii*, CO_2_ availability determines the expression of LHCSR1 and LHCSR3 proteins, the essential protein involved in the photoprotective dissipation of the excitation energy absorbed in excess as heat in a mechanism called non‐photochemical quenching (NPQ): LHCSR1 and LHCSR3 are indeed able to trigger NPQ by both sensing luminal pH and dissipating as heat the excitation energy present in excess in both Photosystem I and Photosystem II (Tokutsu and Minagawa, 2013; Allorent et al., 2013; Peers et al., 2009; Girolomoni et al., 2019; Camargo et al., 2021; Ballottari et al., 2016; Liguori et al., 2016). The expression of LHCSR1 and LHCSR3 is differently modulated by CO_2_ availability: LHCSR1 expression is induced by high CO_2_ availability, while LHCSR3 is downregulated (Maruyama et al., 2014; Yamano et al., 2008; Polukhina et al., 2016; Ruiz‐Sola et al., 2023; Redekop et al., 2022). The effect of CO_2_ availability on NPQ is different in different microalgal species: high CO_2_ was reported to stimulate NPQ in *Chlorella sorokiniana*, while the opposite effect was observed in *Asterarcys quadricellulare* (Varshney et al., 2020).

CO_2_ availability has also been reported to influence the assembly of the external LHC complex to the PSII complex, modulating PSII light‐harvesting efficiency (Berger et al., 2014; Mussgnug et al., 2005; Wobbe et al., 2009; Berger et al., 2016; Blifernez‐Klassen et al., 2021). The transcription factor LCRF (low carbon dioxide response factor) triggers, in conditions of low CO_2_ availability, the accumulation of the cytosolic RNA‐binding protein NAB1 proteins, which represses the translation of mRNA encoding for LHC proteins (Blifernez‐Klassen et al., 2021; Berger et al., 2014; Mussgnug et al., 2005). Consequently, at low CO_2_ availability, the PSII light‐harvesting efficiency is decreased, diminishing the excitation pressure on the photosynthetic apparatus (Berger et al., 2014). This CO_2_ availability‐dependent regulation of PSII light‐harvesting efficiency was not observed in other green algal species, such as *Chlorella vulgaris* or *Chlorella sorokiniana*, even if a NAB1 homolog could be found in *C. vulgaris* (Cecchin et al., 2021).

Even if information was obtained about the adaptation mechanisms of the model green algae *C. reinhardtii* to different CO_2_ availability, few reports specifically addressed the interplay between high light and CO_2_ availability, essentially focused on the investigation of the pattern of LHCSR expression and induction of photoprotective mechanisms. This study investigated the metabolic adaptation in photosynthetic and respiratory processes in *C. reinhardtii* cells grown at low vs. high CO_2_ regimes (3% CO_2_) in non‐limiting light conditions.

## 2 MATERIALS AND METHODS

### 2.1 Microalgae cultivation

*Chlamydomonas reinhardtii* 4A+ strain cells were grown in High‐Salt (HS) medium (Kropat et al., 2011, Harris, 2009) in photoautotrophy in 80 mL air‐lifted photobioreactors in the Multi‐Cultivator MC 1000‐OD (Photon System Instrument) tubes aerated with air (AIR conditions) or with 3% CO_2_‐enriched air (CO_2_ conditions) obtained by a gas mixing system (Gas Mixing System GMS 150, Photon System Instrument). Cells were exposed to different irradiances (100, 200, 500, and 1000 μmol photons m^−2^ s^−1^). After the initial evaluation of the effect of the different irradiances on cell growth, all the experiments were performed at 500 μmol photons m^−2^ s^−1^. Cell density was determined by Countless®II FL automated cell counter (Thermo Fisher). The sampling for the physiologic analysis was done at mid‐exponential phase, and thus with different timing for cells grown in AIR or CO_2_ conditions: after the preliminary experiments where the growth curves of AIR vs. CO_2_ conditions were properly evaluated, the cultivation in these two conditions were conducted with different starting time to allow simultaneous sampling of cells at mid‐exponential phase.

### 2.2 Biomass analysis

Dry weight was determined at the end of the growth curves, and the cells were harvested by centrifugation at 4500 x *g* for 5 minutes at 20°C; the harvested biomass was dried in a lyophilizer for 48 h. Biomass composition was analyzed as previously reported in Cecchin et al. (2019). Briefly, total lipids were extracted from sonicated cells following Bligh and Dyer's method (Bligh and Dyer, 1959). Lipid extracts were separated on thin layer chromatography and quantified for neutral or polar lipids based on densitometry and the comparison to lipid standards (Siaut et al., 2011). The fatty acid composition was determined by converting extracted fatty acids to their fatty acid methyl esters (FAMEs) and then analyzed using a gas chromatography–flame ionization detector (GC‐FID) (Siaut et al., 2011). Proteins content was analyzed by bicinchoninic acid (BCA) protein assay (Thermo Fisher Scientific). Starch content was analyzed by colorimetric reaction with iodine, as previously described (Cecchin et al., 2019).

### 2.3 SDS‐PAGE and immunoblotting

SDS‐PAGE analysis was performed using the Tris‐Tricine buffer system (Schägger and von Jagow, 1987). Immunoblotting analysis were performed upon transfer of SDS‐PAGE gels on nitrocellulose filters. The following antibodies obtained from Agrisera company were used for immunoblotting: α‐PsaA (AS06 172, dilution used 1:3000), α‐PsbC (CP43) (AS11 1787, dilution used 1:2000), α‐AtpC (AS08 312, dilution used 1:10000), α‐RbcL (AS03 037, dilution used 1:5000), α‐Lhcbm5 (AS09 408, dilution used 1:5000), α‐LHCSR1 (AS14 2819, dilution used 1:2000), α‐LHCSR3 (AS14 2766, dilution used 1:2000), α‐CAH3 (AS05 073, dilution used 1:2000). The secondary antibody used was an anti‐rabbit IGG (A3687, Merck) conjugated with alkaline phosphatase as chromogenic detection system.

### 2.4 Pigment analysis

Carotenoids and chlorophylls were extracted in acetone 80% and analyzed by HLPC (Lagarde et al., 2000).

### 2.5 Oxygen evolution, photosynthetic parameters, and NPQ measurements

The light‐dependent oxygen evolution activity of the cultures was measured on samples with a cell density of 3x10^6^ cells mL^−1^ at 25°C with a Clark‐type O2 electrode (Oxygraph Plus, Hansatech) during illumination with light from a halogen lamp (Schott) at different actinic lights (from 50 to 2500 μmol photons m^−2^ s^−1^) in presence of 5 mM sodium bicarbonate. Net oxygen evolution data were obtained upon subtraction of oxygen consumption rate in the dark and fitted with the hyperbolic function y = Pmax * x/(KI + x), where Pmax is the maximum net oxygen evolution rate and KI is the light intensity at which the net oxygen evolution rate is Pmax/2. Photosynthetic parameters ΦPSII, 1‐qL, ETR, and NPQ (Van Kooten and Snel, 1990, Baker, 2008) were measured with a DUAL‐PAM‐100 fluorimeter (Heinz‐Walz) acquiring chlorophyll fluorescence of intact cells on samples with a cell density of 3x10^6^ cells mL^−1^, at room temperature in a 1x1 cm cuvette mixed by magnetic stirring upon exposure to saturating light of 4000 μmol photons m^−2^ s^−1^ and actinic lights from 50 to 2500 μmol photons m^−2^ s^−1^.

### 2.6 Photosystem I activity

P700 activity was measured with the DUAL‐PAM‐100 (Heinz‐Walz) at room temperature in a 1x1 cm cuvette mixed by magnetic stirring on samples with a cell concentration of 1x10^8^ cells mL^−1^ following the transient absorption at 830 nm upon exposure to actinic light from 50 to 1200 μmol photons m^−2^ s^−1^. Maximum P700 activity was measured after a pulse of saturating light (4000 μmol photons m^−2^ s^−1^) in whole cells treated with 50 μM DCMU (3‐(3,4‐chlorophenyl)‐1,1‐dimethylurea), 2 mM sodium ascorbate, and 250 mM methyl‐viologen, as described in Bonente et al. (2012).

### 2.7 NAD(P)H redox state

The formation rate of NAD(P)H was determined with the NADPH/9‐AA module of the Dual‐PAM 101 (Schreiber and Klughammer, 2009), as previously described (Cecchin et al., 2021). Measurement was performed at the same light intensity of growth (500 μmol photons m^−2^ s^−1^) at room temperature in a 1x1 cm cuvette mixed by magnetic stirring on samples with a cell concentration of 1x10^8^ cells mL^−1^. The slope during the exposure to actinic light was used to determine the NAD(P)H formation rate.

### 2.8 Photosystem II light‐harvesting efficiency and state transitions

PSII light‐harvesting efficiency, or functional antenna size, was measured with a DUAL‐PAM‐100 fluorimeter (Heinz‐Walz) from fast chlorophyll induction kinetics induced with a red light of 11 μmol photons m^−2^ s^−1^ (Malkin et al., 1981). Measurements were done on cells concentrated at 3x10^6^ cells mL^−1^ dark‐adapted for 30 minutes and incubated for 5 minutes with 50 μM DCMU. The reciprocal of time corresponding to two‐thirds of the fluorescence rise (τ_2/3_) was considered a proxy for the PSII functional antenna size (Malkin et al., 1981). State transitions were induced in 10^7^ cells with the protocol present in (Drop et al., 2014). In brief, state 1 (S1) was induced by shaking cells in a low light (⁓5 μmol m^2^ s^−1^) with 10 μm of DCMU for at least 15 min to oxidize the plastoquinone pool while state 2 (S2) was induced by adding 250 μm sodium azide to inhibit mitochondrial respiration and to reduce the plastoquinone pool. State transitions were then evaluated by measuring Chl fluorescence emission at 77 K, where both PSI and PSII emission are detectable. 77 K fluorescence emission spectra were acquired with a charge‐coupled device spectrophotometer (JBeamBio) equipped with USB2000+ spectrometer (OceanOptics) and 475 nm LED light sources for excitation. State transitions were estimated as (F_S2_‐F_S1_)/F_S2_, being F_S1_ and F_S2_ the maximum fluorescence emission at 720 nm (PSI emission) measured in cells respectively in S1 or S2.

### 2.9 Electrochromic shift

Proton motive force upon exposure to different light intensities was measured by Electrochromic shift (ECS) according to Kuhlgert et al. (2016). Briefly, cells were dark‐adapted for 20 minutes at a cell concentration of 1x10^8^ cells mL^−1^, then ECS was measured on a 2 mm cuvette with MultispeQ v2.0 (PhotosynQ) at different actinic lights (from 50 to 2400 μmol photons m^−2^ s^−1^). 50 μM DCMU treatment was applied and incubated at least 5 minutes to inhibit linear electron flow: residual ECS was determined to estimate the contribution of cyclic electron flow to ECS formation and calculated as the percentage of ECS signal in DCMU‐treated cells, where linear election flow is inhibited, compared to the untreated ones.

### 2.10 Mitochondrial respiration

Dark respiration was measured on whole cells concentrated at 3x10^6^ cells mL^−1^ and dark‐adapted to 30 minutes using a Clark‐type O_2_ electrode (Oxygraph Plus; Hansatech Instruments). Respiratory rates were normalized to cell density. The individual contributions of the alternative and the cytochrome pathway to mitochondrial respiration were estimated by measuring oxygen consumption in the presence of specific inhibitors: alternative respiration was inhibited by adding 2 mM SHAM (salicyl hydroxamic acid). In contrast, the cytochrome pathway (complex III) was inhibited by adding 5 μM myxothiazol (Bailleul et al., 2015). SHAM and myxothiazol inhibitors were added to whole cells with an incubation time of 5 minutes.

### 2.11 Statistics analysis

All the described experiments were performed at least on two independent lines for each mutant genotype in at least four independent biological experiments. Statistical analysis was performed by using a two‐sided Student's t‐test or ANOVA with post‐hoc Tukey test in the case of multiple comparisons.

## 3 RESULTS

### 3.1 Growth and biomass accumulation

**FIGURE 1:**
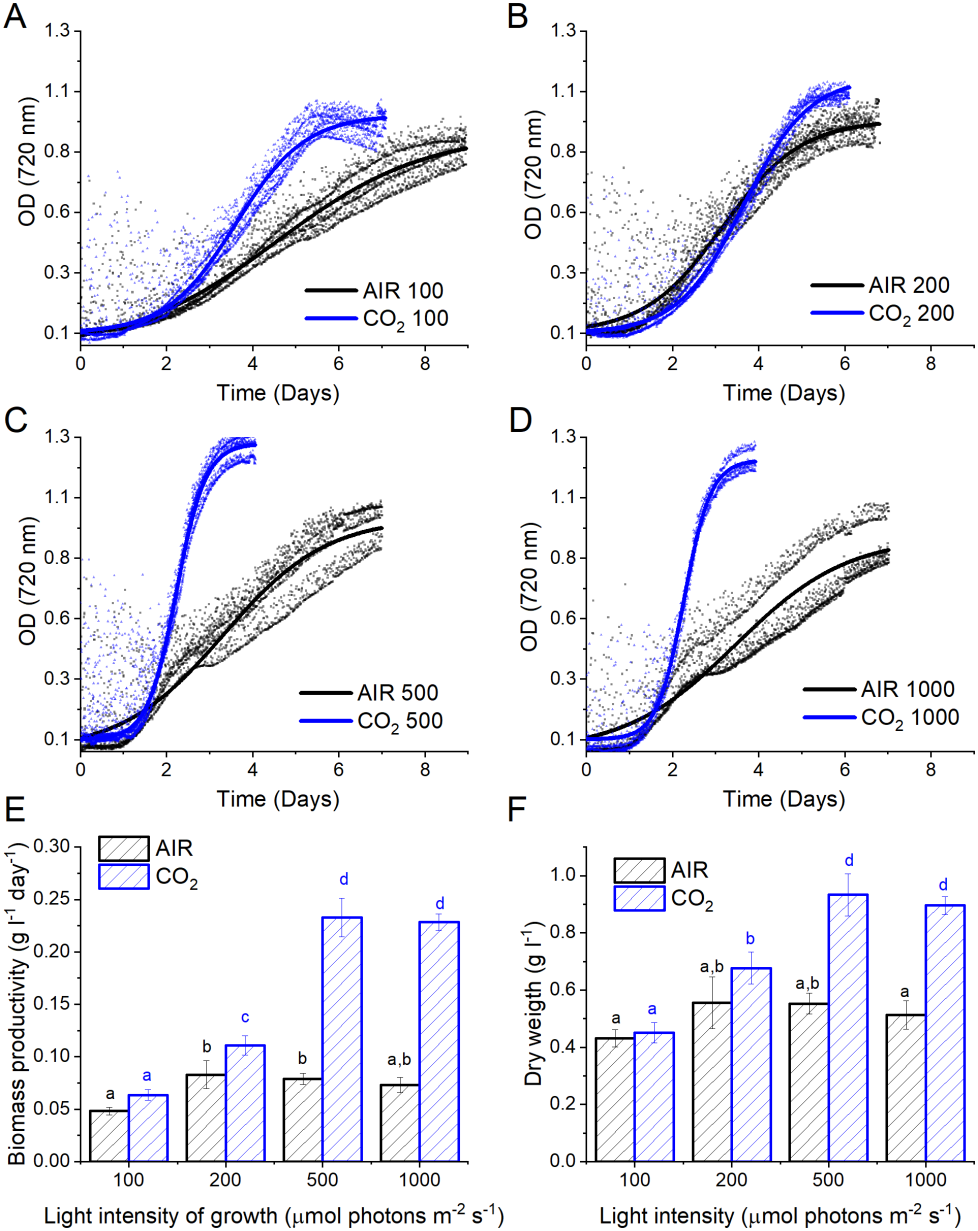
Growth curves and biomass productivity. *Chlamydomonas reinhardtii* cells were grown at different CO_2_ availability (AIR: 0.04%, CO_2_: 3%) at different irradiances (100, 200, 500, or 1000 μmol photons m^−2^ s^−1^ as reported respectively in panels A, B, C, D). In panel A‐D, three different growth curves are reported for each condition, while the functions obtained upon fitting procedure are reported in solid lines. Biomass productivity expressed as g L^−1^ day^−1^ of dry weight and the final biomass (dry weight) concentration are reported in panels E and F. In panels E and F, data are reported as means of three biological replicates with standard deviation shown. Significant different values in the different conditions are indicated by different letters according to ANOVA analysis post Tuckey post‐hoc test (*p* < 0.05).

The influence of carbon availability on *C. reinhardtii* biomass productivity was assessed in photoautotrophic conditions in lab‐scale (80 mL) airlift photobioreactors at different light intensities (100, 200, 500, and 1000 μmol photons m^−2^ s^−1^). Photobioreactors were insufflated with air containing an atmospheric level of CO_2_ (⁓0.04% CO_2_, defined as AIR condition) or air enriched with 3% CO_2_ (defined as CO_2_ condition). PSII maximum quantum yield measured as F_v_/F_m_ was similar in all the conditions tested, comprising 0.6 and 0.7 (Figure S1). F_v_/F_m_ is usually adopted as an indicator of stress in photosynthetic organisms: a similar PSII quantum yield in the different conditions tested in light intensities and CO_2_ availability demonstrates that *C. reinhardtii* can tolerate the growth conditions herein tested. Growth kinetics were determined by measuring cell scattering at 720 nm, and the growth curves were then fitted with sigmoidal functions (shown in Figure 1A‐D). The dry biomass accumulation at the conditions tested was consistent with the different cell concentrations measured at the end of the growth curves (Figure S2). Cells grown in CO_2_ present a faster growth rate than the AIR condition, as shown by the first derivative of the growth curves (Figure S3) and the calculated average daily biomass productivity (Figure 1E). Biomass accumulation was generally higher in CO_2_ compared to AIR conditions. In AIR conditions, in terms of dry weight, the biomass concentration achieved at the end of the growth ranges from ~0,4 to ~0,5 g/L with similar values at light intensities above 200 μmol photons m^−2^ s^−1^. In the CO_2_ condition, biomass productivity ranged from ~0,4 to ~0,9 g/L with an increasing biomass accumulation and daily productivity at higher irradiances being saturated only above 500 μmol photons m^−2^ s^−1^ (Figure 1F). These results suggest that CO_2_ availability represented the limiting factor in AIR, while light intensity was the limiting factor in CO_2_ conditions. According to these results, the following analyses focused on samples grown at 500 μmol photons m^−2^ s^−1^, the lowest irradiance where the growth was saturated in both AIR and CO2 conditions. Moreover, the most significant difference in biomass accumulation between AIR and CO2 conditions was observed at 500 μmol photons m^−2^ s^−1^.

### 3.2 Biomass composition and lipids accumulation

**FIGURE 2:**
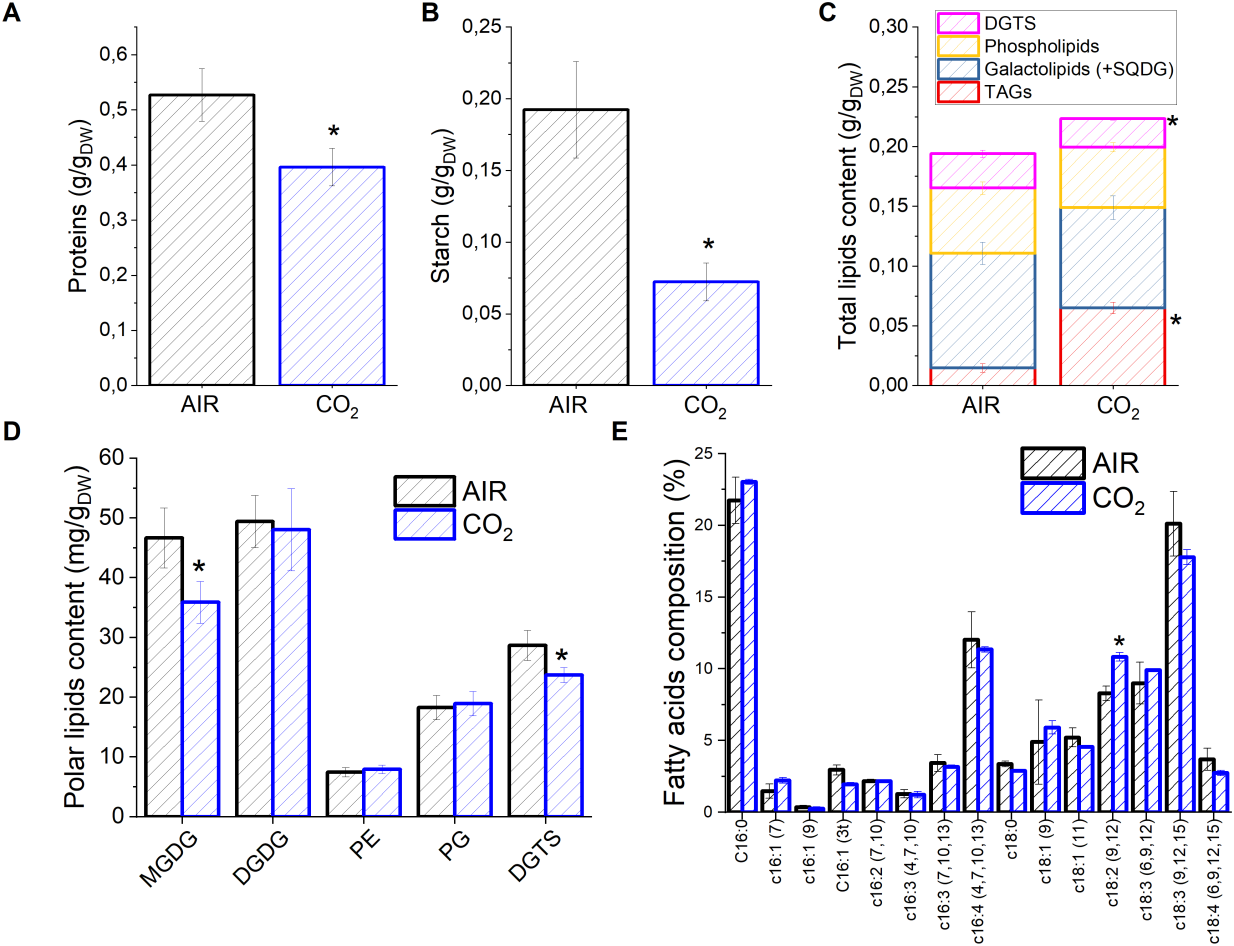
Protein, starch, and lipids in AIR *vs*. CO_**2**_ conditions. Relative protein (A), starch (B), and lipid (C) content per dry weight in AIR *vs*. CO_2_ condition. Lipid composition is reported in terms of phospholipids, galactolipids (including SQDG), DGTS, and triacylglycerols (TAG). (D) Polar lipid profile obtained by thin‐layer chromatography. (C) The profile of fatty acids was obtained by gas chromatography. Data are means of three biological replicates with standard deviation shown. Significantly different values in CO_2_ versus AIR are indicated with * (*p* < 0.05, *n* = 3). MGDG, monogalactosyldiacylglycerol; DGDG, di galactosyl diacylglycerol; PG, phosphatidylglycerol; PE, phosphatidylethanolamine; DGTS, diacylglycerol *N,N,N*‐trimethylhomoserine; SQDG, sulfoquinovosyl diacylglycerol.

At the end of the growth experiment, the biomass produced by cells grown at 500 μmol photons m^−2^ s^−1^ was sampled to evaluate its composition, analyzing the main carbon sinks as starch, proteins, and lipids (Figure 2). Specifically, the fractions of starch and proteins on cell dry weight were lowered in a CO_2_‐enriched regime compared to AIR (Figure 2A,B). In the case of lipids, their total content increased in CO_2_ conditions, with a significantly different composition. Indeed, among lipid classes, triacylglycerol (TAG) levels were strongly increased at high CO_2_ availability (Figure 2C). The polar lipids' compositional analysis evidenced a decreased amount of MGDG galactolipids fraction in the CO_2_ condition, being MGDG the principal constituent of the thylakoid membranes (Kobayashi, 2016; Aronsson et al., 2008). High CO_2_ availability also led to a slight but significant decrease of the betaine lipid diacylglycerol N, N, N‐trimethyl homoserine (DGTS), a lipid found in the endoplasmic reticulum in *C. reinhardtii* (Moore et al., 2001) and previously reported to be a potential source of fatty acid for TAG biosynthesis (Valledor et al., 2014; Yang et al., 2020). TAGs surplus registered in CO_2_ may derive both from *de novo* lipid biosynthesis and recycling of pre‐existent membrane lipids (Simionato et al., 2013), which in this case could probably originate mainly from the thylakoids' MGDG galactolipids and DGTS. Fatty acids distribution was then analyzed in detail: in both cases, AIR and CO_2_ samples, the primary fatty acids observed were palmitic acid (C16:0), linoleic acid (C18:2^9,12^), α‐linolenic acid (C18:3^9,12,15^), γ‐linolenic acid (C18:3^6, 9, 12^) and hexadecatetraenoic acid (C16:4^4,7,10,13^). While most of the fatty acids were similarly accumulated in AIR vs. CO_2_ conditions, the only significant difference was observed in the case of linoleic acid (C18:2^9,12^), whose relative accumulation among fatty acids was increased in CO_2_ conditions (Figure 2E). Interestingly, linoleic acid was previously observed to be positively correlated to TAG amount, e.g., present in higher amounts when TAG was increased in *C. reinhardtii* under nitrogen starvation (Valledor et al., 2014).

### 3.3 NAD(P)H redox state and respiration

**FIGURE 3:**
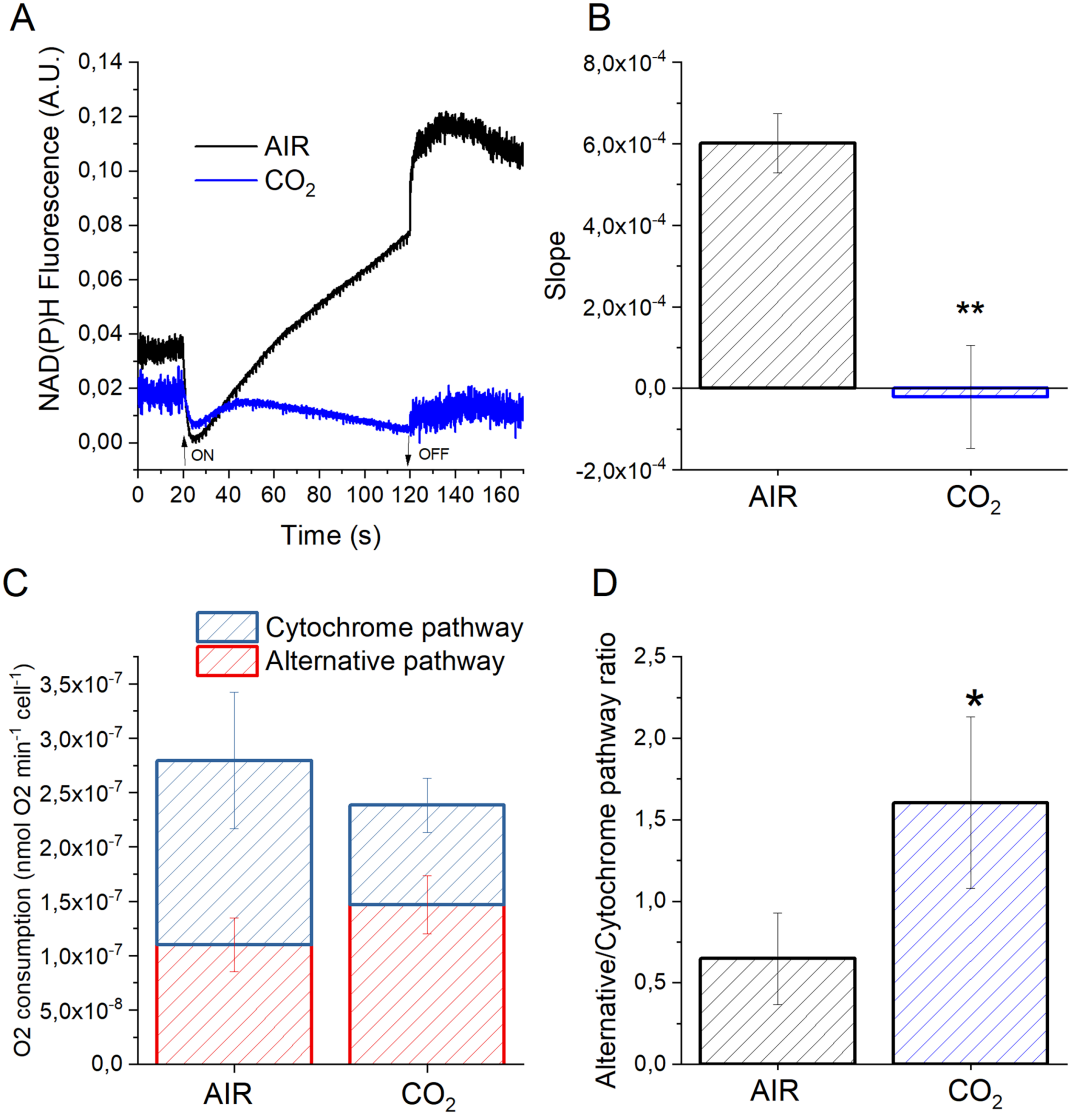
NAD(P)H formation rate and mitochondrial respiration in AIR *vs*. CO_**2**_. (A) Light‐dependent rate of NAD(P)H formation upon exposure to light (300 μmol photons m^−2^ s^−1^) for 100 s in AIR (black color) or CO_2_ (blue color) conditions followed by 60 s of dark recovery (ON–OFF in panel A indicates when light was turned on or off). (B) Slope of the NAD(P)H fluorescence emission curve upon exposure to actinic light reported in (A). (C) Oxygen consumption by dark respiration in cells grown in AIR or CO_2_ conditions. The relative contribution of cytochrome (filled bars) and alternative respiration (empty bars) was reported normalized to cell content. (D) The ratio between alternative and cytochrome pathways for cells grown in AIR or CO_2_ conditions according to the results reported in (C). Data are means of three biological replicates with standard deviation shown. Significantly different values in CO_2_ versus AIR are indicated by * (*p* < 0.05) or ** (*p* < 0.01).

Carbon fixation and fatty acid biosynthesis require reducing power. NAD(P)H formation and the respiratory metabolism were thus investigated. Specifically, light‐dependent NADPH formation was evaluated by following NAD(P)H fluorescence changes upon exposure to 500 μmol photons m^−2^ s^−1^ light for 120 seconds (Figure 3A). In the AIR condition, the NAD(P)H accumulation rate is positive, meaning NAD(P)H production exceeds the consumption rate. On the other hand, in the CO_2_ condition, the formation's slope is almost zero, which means that the NAD(P)H accumulation rate equals the consumption one (Figure 3B). These results are consistent with the increased requirement of NADPH in the case of cells grown in CO_2_ conditions to provide reducing power required for CO_2_ assimilation. NADH consumption by mitochondrial respiration was evaluated using a Clark‐type electrode in the dark. Mitochondrial respiration produces ATP while releasing NAD^+^ via the respiratory electron transport, including an ATP synthase complex and four oxidoreductase complexes. Electron transport produces an electrochemical gradient across the mitochondrial membranes, which drives oxidative phosphorylation. An alternative oxidase (AOX) catalyzes ubiquinol oxidation coupled to O_2_ reduction to H_2_O, generating an alternative branch for the electron flow of the mitochondrial transport chain. AOX activity dramatically decreases ATP yields per NADH, acting as an overflow protection mechanism of the respiratory chain (Vanlerberghe, 2013; Boekema and Braun, 2007). Dark respiration rates were essentially unaffected by different CO_2_ availabilities (Figure 3C). The individual contributions of the cytochrome and alternative pathways were explored by measuring dark respiration upon inhibition of AOX with SHAM (salicyl hydroxamic acid) or complex III with myxothiazol, blocking the alternative and the cytochrome pathway, respectively (Zhang et al., 2012; Cecchin et al., 2023). The relative contribution of cytochrome and alternate pathways to the total respiration rate differed in the two growth conditions, with the cytochrome pathway being disadvantaged in favor of the alternative one in CO2 conditions (Figure 3D).

### 3.4 Pigments and photosynthetic protein accumulation

**FIGURE 4:**
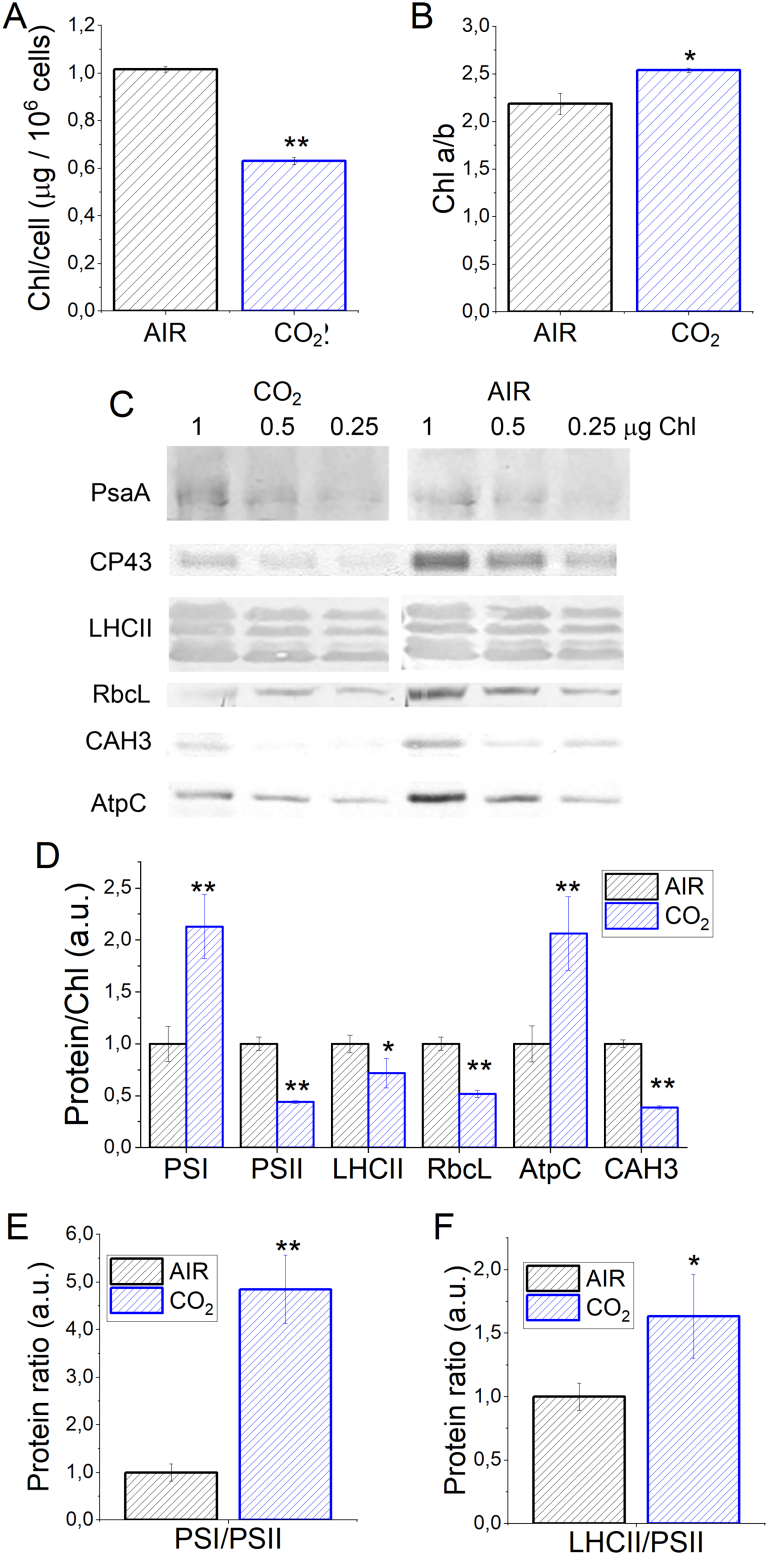
Pigment and photosynthetic protein accumulation. (A) Chlorophyll (Chl) content per cell in AIR or CO2 conditions. (B) Chlorophyll *a*/*b* ratio in AIR or CO_2_ conditions. (C) Immunoblotting results of different proteins involved in the photosynthetic process: Photosystem I subunit PsaA, Photosystem II subunit CP43, LHCII antenna proteins, RuBisCO large subunit (RbcL), chloroplastic ATPase subunit C (AtpC) and carbonic anhydrase (CAH3). (D) densitometric analysis of western blot reported in (C) expressed as protein content on a chlorophyll (Chl) basis normalized to AIR condition. (E) The Photosystem I (PSI) to Photosystem II (PSII) ratio is calculated based on the results reported in (D). (F) LHCII to Photosystem II (PSII) ratio calculated based on the results reported in (D) normalized to AIR condition. Data are means of three biological replicates with standard deviation shown. Significantly different values in CO_2_ versus AIR are indicated by * (*p* < 0.05) or ** (*p* < 0.01).

NADPH formation in photosynthetic organisms is strongly related to their photosynthetic activity. Accumulation of pigments and photosynthetic proteins were thus investigated. Chlorophyll (Chl) content per cell was reduced in the CO_2_ conditions (Figures 4A and S4), while the Chl *a*/*b* ratio significantly increased (Figure 4B). Chl *b* is bound exclusively to the external antenna complexes of PSI and PSII, the LHCs complexes. In contrast, Chl *a* is bound to both LHCs and Photosystem core complexes: different Chl *a*/*b* thus suggests possible reorganization of antennae and core complexes relative stoichiometries. Alternatively, the relative Chl *a* content of PSI being higher than PSII, a different Chl *a*/*b* ratio might be related to different PSI/PSII ratios. The relative content of the PSI, PSII, and LHCII proteins was thus investigated by immunoblot (Figure 4C). PSI content was significantly increased on a Chl basis in CO_2_ conditions compared to AIR samples, while the opposite was observed for PSII (Figure 4D). Consequently, a substantial PSI/PSII ratio increase was evident in CO_2_ (Figure 4E). In the case of LHCII, it is worth noting that the antibody adopted, α‐Lhcbm5, was previously reported to recognize all the different Lhcbm subunits encoded by the *C. reinhardtii* genome (Girolomoni et al., 2017). LHCII content on a Chl basis decreased in CO_2_ compared to AIR conditions (Figure 4D), which aligns with a general decrease in PSII subunits. However, the LHCII/PSII ratio was increased at high CO_2_ availability (CO_2_ conditions), suggesting a possible increase in PSII light‐harvesting properties (Figure 4F). The growth in presence of low CO_2_ availability (AIR condition) is expected to induce the carbon concentrating mechanism (CCM), a complex set of regulatory elements and enzymes involved in actively concentrating inorganic forms of carbon inside of the cell, precisely close to the site of fixation, the RuBisCO enzyme (Wang et al., 2015). The accumulation of RUBISCO and carbonic anhydrase, a component of CCM, were thus investigated by immunoblot: on a cell basis, in CO_2_‐limiting conditions (AIR), both the RuBisCO and carbonic anhydrase content were increased (Figures 4 and S5), while chloroplastic ATPase was increased in cells grown in CO_2_ conditions (Figure 4C, D). These findings indicate that carbon availability triggers specific adaptation of the photosynthetic apparatus in terms of the light and metabolic phases and CCM mechanisms.

This increased RuBisCO content observed in AIR vs. CO_2_ conditions differs from previous findings in the case of *C. vulgaris* or *C. sorokiniana*, where CO_2_ availability was reported not to affect RuBisCO accumulation (Cecchin et al., 2021); however, it is essential to note that, in the case of *C. vulgaris* and *C. sorokiniana*, the evaluation of the effect of CO_2_ availability on cell physiology was done only at a light intensity saturating the photosynthetic activity of the cells (500 μmol photons m^−2^ s^−1^). Accordingly, no significant change was observed when RuBisCO content was investigated in *C. reinhardtii* grown at lower light intensities (100 or 200 500 μmol photons m^−2^ s^−1^) on a Chl or cell basis (Figure S6).

### 3.5 Light‐harvesting and state transitions

**FIGURE 5:**
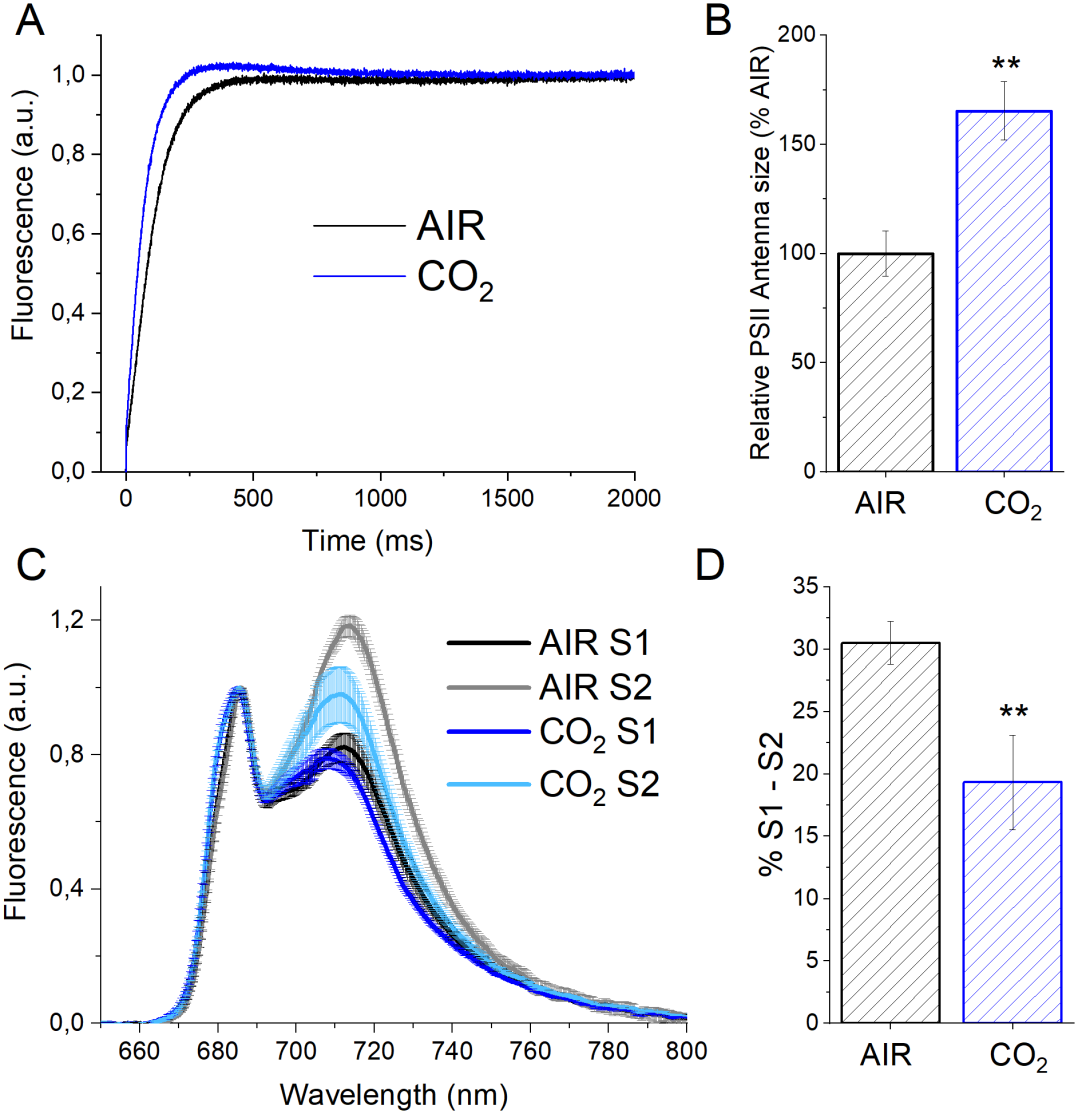
Photosystem II light harvesting efficiency and state transitions in AIR *vs*. CO_2_. (A). fluorescence emission kinetic upon exposure to limiting light in cells treated with DCMU to inhibit PSII photochemical activity. (B) PSII light harvesting efficiency, or PSII antenna size, calculated from the kinetics reported in (A) as the 1/τ_2/3_, where τ_2/3_ is the time required to reach 2/3 of the maximum fluorescence emission. The PSII light harvesting efficiencies were then normalized as a percentage of light harvesting efficiency measured in cells grown in AIR conditions. (C) State transition analysis by 77 K fluorescence emission spectra in state 1 (S1) or state 2 (S2) conditions. S1 was induced by shaking vigorously cells in a low light (⁓5 μmol photons m^2^ s^−1^) with 10 μm of DCMU for at least 15 min to oxidize the plastoquinone pool. In contrast, S2 was induced by adding 250 μm sodium azide to inhibit mitochondrial respiration and to reduce the plastoquinone pool. (D) Maximum capacities for state transitions estimated from the spectra reported in (C) as (F_S2_‐F_S1_)/F_S2,_ being F_S1_ and F_S2_ the maximum fluorescence emission at 720 nm (PSI emission) measured in cells respectively in S1 or S2. Data reported are means of three biological replicates with standard deviation shown. Significant differences in CO_2_ *vs*. AIR are indicated with ** (*p* < 0.01).

To investigate if the increased LHCII/PSII in conditions of high CO_2_ availability caused increased light‐harvesting properties of PSII, the functional PSII antenna size was analyzed by measuring Chl *a* fluorescence induction curve upon exposure to limiting light in the presence of PSII inhibitor DCMU (Figure 5A). In this condition, the kinetic of the fluorescence emission curve is inversely proportional to the PSII antenna size. The registered increase in functional antenna size in the CO_2_ conditions is around 60% compared to the control (Figure 5B); this result is consistent with the LHCII/PSII ratio estimated by the western blot (Figure 4C). Such outcome is consistent with previous reports and ascribed to the effects of NAB1 protein, accumulated in CO_2_‐limiting conditions in *C. reinhardtii*: NAB1 acts as regulatory hub, controlling PSII excitation pressure in function of the carbon availability by repressing the translation of specific transcripts, LHC subunits included (Blifernez‐Klassen et al., 2021, Berger et al., 2014, Cecchin et al., 2021).

The capability of cells grown in CO_2_ or AIR conditions to perform state transitions was then analyzed. LHCs are partly bound to PSI or PSII. To ensure the excitation balance of the two photosystems, LHC complexes are subjected to a process defined as state transitions. In *C. reinhardtii*, phosphorylation of the LHC complexes using STT7 kinase induces LHC to move from PSII to PSI in the so‐called state 2 (S2). State 1 (S1), in which LHCs move from PSI to PSII, is achieved using a phosphatase (Lemeille et al., 2009; Depege et al., 2003; Lemeille et al., 2010). S1 and S2 can be induced and measured by exploiting Chl fluorescence emission at 77 K (Figure 5C), a situation in which the photochemical activity of the Photosystem is blocked, and both PSI and PSII fluorescence contributions are detectable. Maximum capacities for state transitions were then quantified from the maximum fluorescence emission in the PSI peak (~720 nm) as (F_S2_‐F_S1_)/F_S2_. As shown in Figure 5D, a high CO_2_ regime decreased the capacity of *C. reinhardtii* to realize state transitions, suggesting that a smaller fraction of LHC moiety is available to migrate from PSII to PSI. Both the reduced capacity to switch to State II and the increased PSII antenna size in CO_2_ conditions indicate a general increased requirement of excitation pressure on PSII to maintain a steady linear electron flow of electrons given the increased need for the photosynthetic machinery of NADPH to operate CO_2_ fixation.

### 3.6 Photosynthetic activity

**FIGURE 6:**
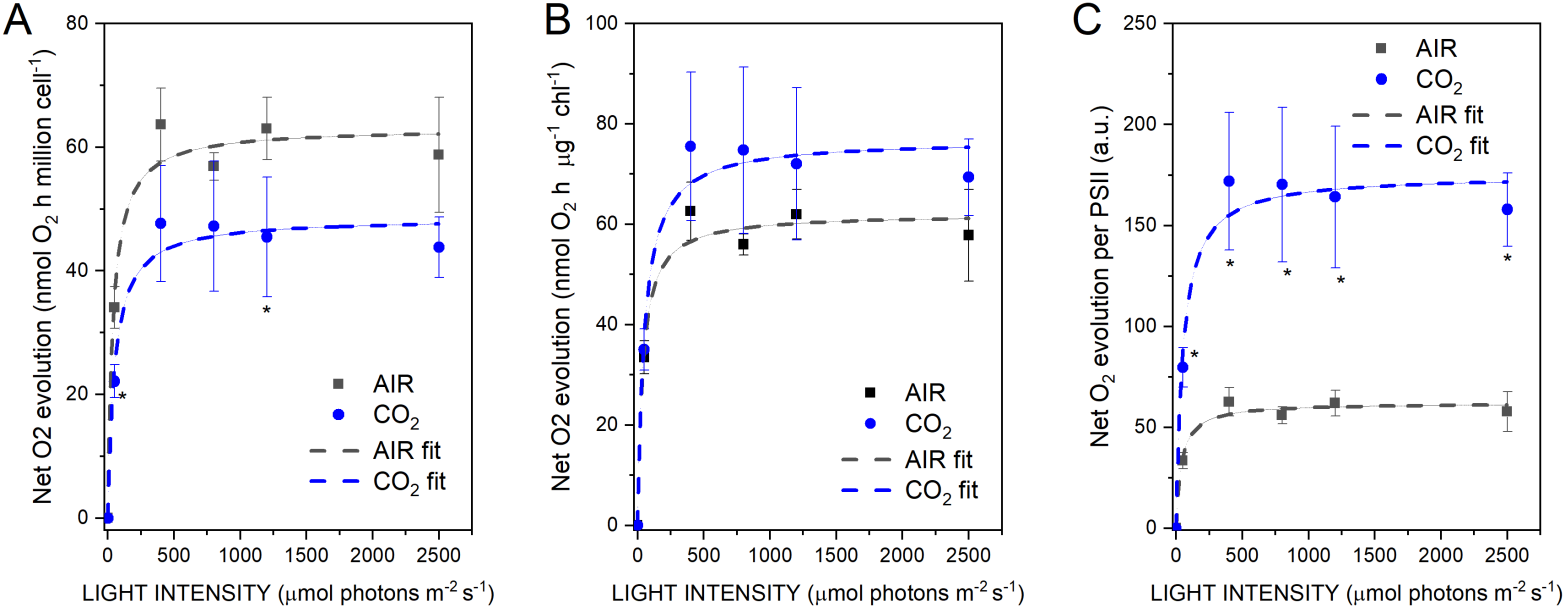
Light‐dependent oxygen evolution curves. Light‐dependent oxygen evolution was measured at different light intensities. The results obtained are presented on a cell basis (A), chlorophyll basis (B), or PSII basis (C). Data reported are means of three biological replicates with standard deviation shown. Significant differences in CO_2_ *vs*. AIR are indicated with * (*p* < 0.05).

The photochemical activity of PSII was then analyzed by measuring light‐dependent oxygen evolution at different actinic lights. Oxygen evolution on a cell basis was generally lower in CO_2_ samples compared to cells grown in AIR conditions, consistently with lower Chl content per cell observed in the former (Figure 6A). Instead, on a Chl basis, slightly higher oxygen evolution was observed in CO_2_ samples (Figure 6B). However, it is essential to note that PSII content was significantly reduced on a Chl basis in cells grown in CO_2_ conditions (Figure 4C): light‐dependent oxygen production on a PSII basis was consequently significantly increased in CO_2_ conditions (Figure 6C).

**FIGURE 7:**
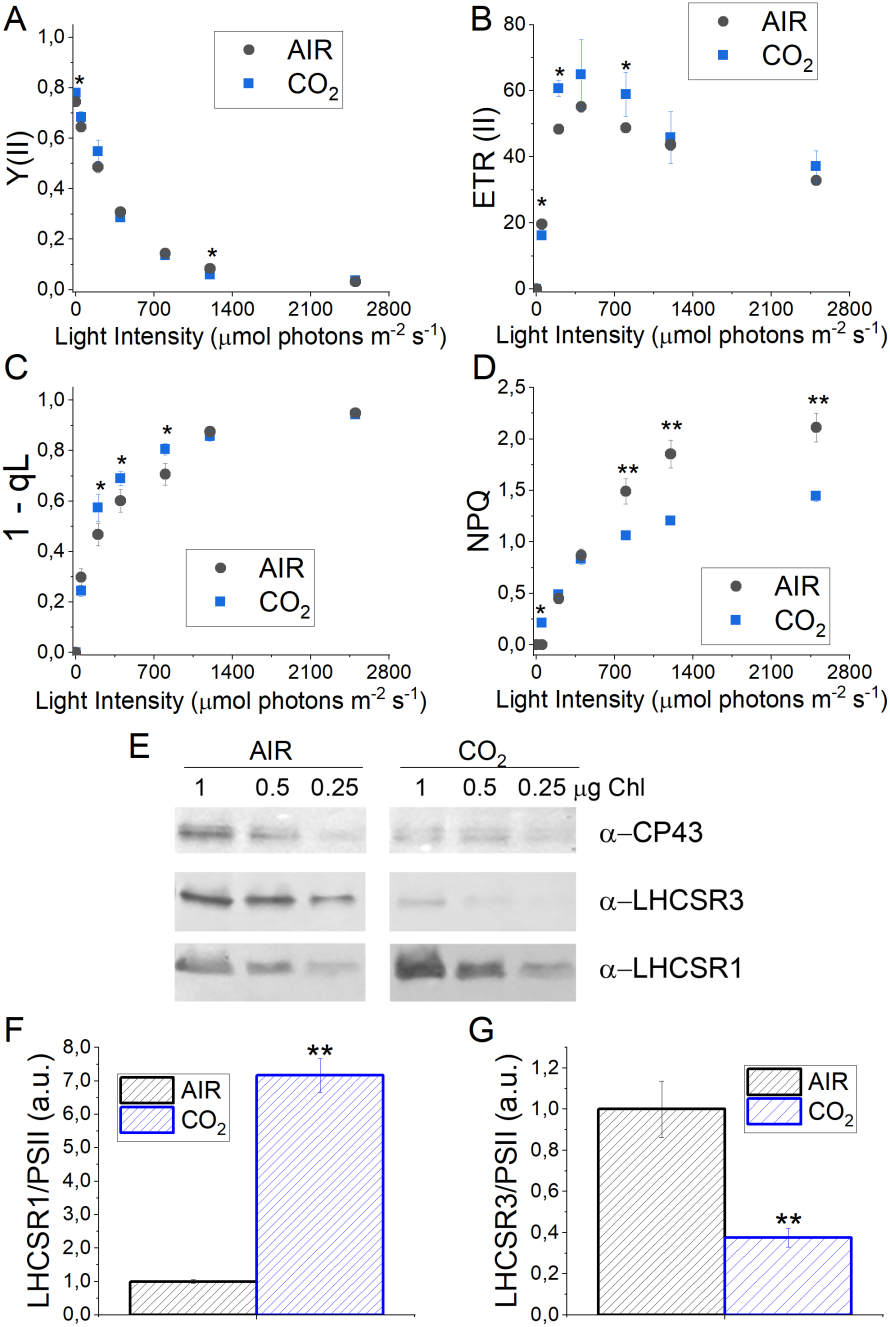
Photosystem II photochemical and non‐photochemical activity. (A) PSII operating quantum yield (Y(II)), (B) PSII electron transport rate (ETR(II)), (C) redox state of plastoquinone (1‐qL), D) non‐photochemical quenching (NPQ) measured at different actinic lights in dark‐adapted cells grown in AIR or CO_2_ conditions. (E) Western blot analysis of LHCSR3 and LHCSR1 content in cells grown in AIR or CO_2_ conditions. CP43 subunit content was also analyzed by immunoblotting to normalize LHCSR1 and LHCSR3 content to PSII. (F) LHCSR1 to PSII ratio determined by densitometric analysis of immunoblotting results (E) normalized to AIR condition. (G) LHCSR3 to PSII ratio determined by densitometric analysis of immunoblotting results normalized to AIR conditions (E). Data reported are means of three biological replicates with standard deviation shown. Significant differences in CO_2_ *vs*. AIR are indicated with * (*p* < 0.05) or ** (*p* < 0.01).

**FIGURE 8:**
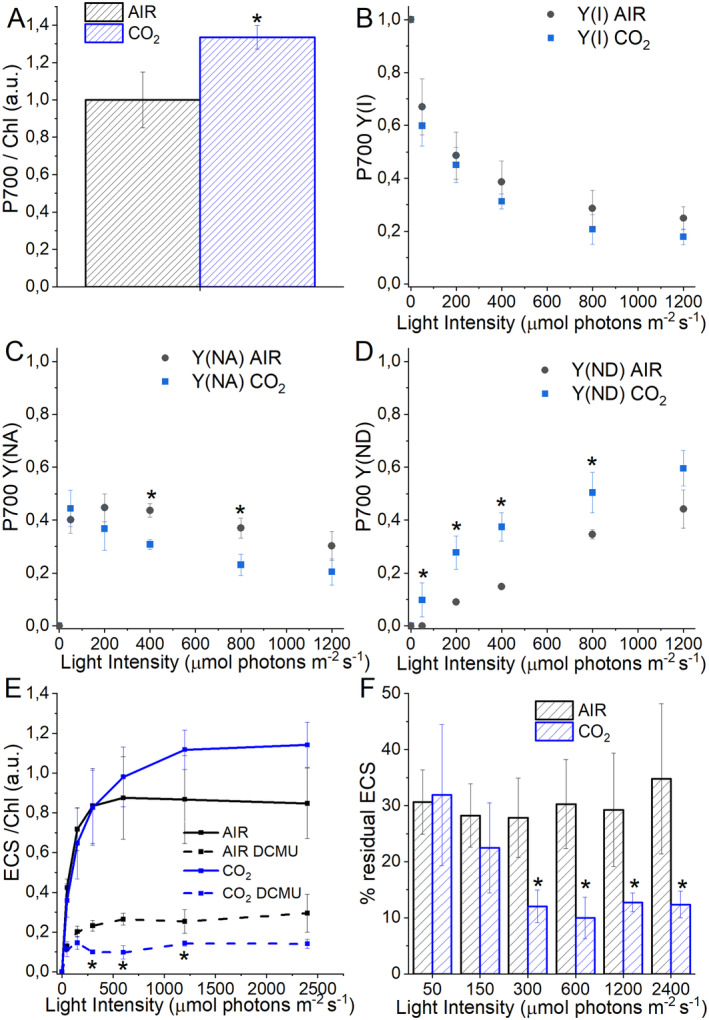
Photosystem I activity and electrochromic shift in AIR vs. CO_2_ condition. (A) Maximal P700 oxidation on a chlorophyll basis in cells grown in AIR or CO_2_ conditions normalized to AIR condition. (B) P700 quantum yield at different actinic lights. (C) Fraction of P700 being limited at the acceptor side at different actinic lights. (D) The fraction of P700 is limited on the donor side at different actinic lights. (E) Electrochromic shift (ECS) at different actinic light in cells grown in AIR or CO_2_ conditions (solid lines) normalized to chlorophyll content. ECS results in the presence of DCMU are also reported with dashed lines. (F) Percentage of residual ECS upon DCMU treatment and inhibition of linear electron flow. Data are means of three biological replicates with standard deviation shown. Significant differences in CO_2_ *vs*. AIR are indicated with * (*p* < 0.05) or ** (*p* < 0.01).

Photosynthetic parameters such as PSII operating quantum yield (Y(II)), electron transport rate (ETR(II)), and redox state of plastoquinone (1‐qL) were then measured. While Y(II) was essentially unchanged in CO_2_ vs. AIR cells (Figure 7A), samples grown in CO_2_ conditions were characterized by increased ETR(II) and increased 1‐qL at light intensities between 200 and 800 μmol photons m^−2^ s^−1^ (Figure 7B, C). These results are consistent with the oxygen evolution data on a PSII basis, suggesting an increased linear electron flow at high CO_2_ availability, likely because of increased NADP+ availability as an electron acceptor, being more efficiently regenerated by the Calvin‐Benson cycle. The induction of NPQ was then measured, being NPQ the photoprotective mechanism by which a fraction of the light energy absorbed is safely dissipated as heat. As reported in Figure 7D, similar NPQ induction was measured for cells grown in AIR or CO_2_ conditions at light intensities below 700 μmol photons m^−2^ s^−1^. In contrast, at higher light intensities, cells growing in CO_2_ conditions were characterized by decreased NPQ induction. NPQ in *C. reinhardtii* has been previously reported to be strongly related to LHCSR1 and LHCSR3 accumulation (Peers et al., 2009), which are differently triggered based on CO_2_ availability (Maruyama et al., 2014). As evidenced by the blot analysis (Figure 7E), the accumulation of LHCSR1 in CO_2_ conditions is increased, while LHCSR3 is decreased (Figure 7F, G). These results are consistent with previous data in the literature with high CO_2_ availability inhibiting LHCSR3 accumulation but stabilizing LHCSR1 expression (Maruyama et al., 2014; Yamano et al., 2008; Polukhina et al., 2016; Ruiz‐Sola et al., 2023; Redekop et al., 2022). PSI photochemical activity was then measured by transient absorption of the oxidized PSI reaction center at 830 nm. In the presence of DCMU, which inhibits electron transport from PSII to PSI, ascorbate (AS) as an electron donor, and methyl viologen (MV) as an electron acceptor, it is possible to determine the maximum P700 activity. A significant increase in the P700 MAX in CO_2_ compared to the AIR conditions was observed (Figure 8). These data are consistent with the results obtained by western blot analysis on subunits of PSI (PsaA) and PSII (CP43). In the absence of inhibitors, P700 activity represents the physiologic photochemical activity of PSI as a balance between electron acceptors and electron donor availability. A similar photochemical yield of PSI (Y(I)) could be measured in AIR and CO_2_ conditions, decreasing at higher actinic light as a consequence of the onset of limitations at both acceptor [Y(NA)) and donor (Y(ND)] sites. However, in the case of AIR cells, increased acceptor side limitation was measured at light intensities higher than 200 μmol photons m^−2^ s^−1^, while increased donor side limitation was measured in CO_2_ condition. These findings are consistent with the increased NADP^+^ availability in CO_2_ condition, as electrons acceptor from ferredoxin and thus from PSI, and with the increased PSI/PSII ratio measured in CO_2_ samples.

The photochemical activity of PSI and PSII drives the photosynthetic electron transport coupled with proton transport across the thylakoid membranes. Electrochromic shift (ECS) of carotenoid absorption can be adopted to estimate the proton‐motive force (*pmf*) generated by the photosynthetic electron transport (Bailleul et al., 2010). Increased ECS values were measured for both AIR and CO_2_ cells at increasing light intensities but were not significantly different between the samples grown at different carbon availability, suggesting a similar induction of *pmf* (Figure 8E). ECS was then measured upon treatment with the PSII inhibitor DCMU: in the presence of DCMU, linear electron flow is blocked, and the *pmf* induced is essentially only related to cyclic electron flow (CEF) to ECS of carotenoid absorption. It is important to note that the evaluation of CEF by inhibiting PSII activity could be underestimated because, in previous works, lower CEF was measured in PSII‐inhibited cells compared to non‐treated cells (Lucker and Kramer, 2013). In the presence of DCMU, a lower residual ECS was measured in the case of cells grown at high CO_2_ availability at measuring light intensities higher than 300 μmol photons m^−2^ s^−1^ (Figure 8A, F). These results suggest that the CEF contribution to the overall photosynthetic proton transport is higher in AIR compared to CO_2_ conditions. This result is consistent with the increased linear electron transport in CO_2_ samples evinced from increased water stripping and oxygen evolution on a Chl and PSII basis. Notably, in CO_2_ cells, an increased ATPase content on a Chl basis was measured (Figure 4C, D), suggesting an increased capability in managing the protons transported through the photosynthetic electron transport chain to synthesize ATP.

## 4 DISCUSSION

Photosynthetic biomass production requires the availability of light, CO_2_, and nutrients. In the case of the experimental conditions herein applied to *C. reinhardtii* cell cultures, maximum biomass productivity was observed at high CO_2_ availability and irradiance above 500 μmol photons m^−2^ s^−1^ (Figure 1). At lower light intensities, biomass productivity was similar in CO_2_ vs. AIR conditions, losing the advantage of providing additional inorganic carbon (Figure 1). Thus, CO_2_ availability becomes the limiting factor for algal biomass accumulation only if sufficient light is provided. Biomass productivity increased in CO_2_ conditions, and biomass composition was influenced by different carbon availability: a decrease in starch accumulation and an increase in TAGs were detected in CO_2_ vs. AIR conditions, suggesting a shift of energy‐storing molecules towards more energy‐dense compounds. Moreover, a change in the relative composition of lipids in the CO_2_ conditions was observed, with a decrease in the content of galactolipids (specifically MGDG), the main lipids of photosynthetic membranes (Li‐Beisson et al., 2019) in favor of TAGs. TAGs could derive from recycling other lipids, such as MGDG or *de novo* biosynthesis (Simionato et al., 2013; Li‐Beisson et al., 2019). Due to decreased starch accumulation, it is possible to suggest that photosynthates are re‐directed toward fatty acids biosynthesis in the chloroplast and ultimately exported in the cytosol for TAGs accumulation (Yang et al., 2013; Valenzuela et al., 2012). Also, an elevated CO_2_ level could directly impact the cellular C/N ratio, diluting the amount of protein within the organism as a response. Indeed, the protein fraction of dry weight was decreased in CO_2_ conditions, likely due to increased CO_2_ assimilation and an increased cellular C/N ratio. Plants degrade RuBisCO as the primary storage protein and nitrogen source during leaf senescence (Diaz et al., 2008). Consistently, the main soluble protein complex in *C. reinhardtii*, RuBisCO, is strongly reduced at high CO_2_ availability but only when grown at saturating light conditions: it cannot be excluded that the increased RuBisCO content in AIR vs. CO_2_ conditions upon high light exposure could be related to adaptation mechanisms to improve regeneration of NADP^+^ even in conditions of carbon limitation, thus allowing to partially desaturate the photosynthetic electron transport chain. In cells grown in AIR conditions, a substantial increase of carbonic anhydrase content per cell was measured (Figure 5), indicating the activation of CCM due to reduced carbon availability, as previously reported in *C. reinhardtii* cultivated in CO_2_‐limiting conditions (Renberg et al., 2010; Xiang et al., 2001; Yamano et al., 2008). Recently, CCM was reported to be dependent on ATP produced by alternative photosynthetic electron transports (Burlacot et al., 2022): accordingly, in the AIR condition, an increased contribution of CEF to the overall pmf was measured, while in the CO_2_ condition, increased LEF was evident (Figure 8). Increased CEF upon limiting CO_2_ availability is also consistent with previous findings by Lucker and Kramer (2013). In CO_2_ samples, the relative increase in LEF is consistent with increased PSII activity, measured as increased ETR and increased light‐dependent oxygen evolution on a PSII basis (Figures 6 and 7). PSII activity requires proper light‐harvesting to fuel its photochemical activity: in CO_2_ conditions, higher LHCII/PSII ratio and improved light‐harvesting efficiency were measured (Figure 5) compared to AIR samples. This observation fits with the regulatory effects of NAB1 as a translational repressor for LHCII encoding mRNAs previously reported to be downregulated in CO_2_ condition, leading to increased accumulation of LHCII subunits (Berger et al., 2014; Cecchin et al., 2021; Berger et al., 2016; Mussgnug et al., 2005). It is also important to note that high CO_2_ availability caused a decrease in NPQ induction (Figure 7). Decreased NPQ at higher actinic lights results from the differential expression patterns of LHCSR isoforms in response to the CO_2_ concentration: high CO_2_ availability caused a lower accumulation of LHCSR3 and higher content of LHCSR1 (Polukhina et al., 2016; Maruyama et al., 2014; Ruiz‐Sola et al., 2023) but the overall effect was a reduced capability to induce NPQ. This finding is consistent with the proposed different role of LHCSRs subunit in tuning NPQ, with LHCSR3 being more efficient than LHCSR1 (Perozeni et al., 2020), even if the absolute comparison of LHCSR3 and LHCSR1 content in AIR and CO2 conditions cannot be adequately evaluated by immunoblotting and requires additional research effort. Nevertheless, decreased NPQ induction in CO_2_ conditions might be a specific cell adaptation that increases excitation pressure on PSII to support increased linear electron flow.

Photosynthetic adaptation to a high CO_2_ regime increases PSI/PSII ratio (Figure 4) and higher P700 activity on a Chl basis (Figure 8). PSI is responsible for the light‐dependent electron transport from plastocyanin to ferredoxin and then to NADPH: the increased carbon availability in CO_2_ conditions likely increases NADP^+^ availability, inducing a higher demand for electrons from PSI to produce NADPH. Accordingly, the photochemical activity of PSI in CO_2_ samples was mainly limited on the donor side. On the contrary, in AIR conditions, PSI was mainly limited on the acceptor side (Figure 8), likely due to a lower availability of NADP^+^.

The results of the NADPH/NADP+ balance demonstrate that NAD(P)H formation in cells grown at high CO_2_ availability was balanced with its consuming rate. In contrast, in the AIR condition, NAD(P)H net accumulation was observed (Figure 3). It is important to note that the fluorescence‐based measurement reported in Figure 3 cannot distinguish between NADPH and NADH. According to these findings, it is possible to hypothesize that increased CO_2_ availability determines a higher NAD(P)H consumption in cells grown in CO_2_ condition, which matches the rate of NAD(P)H formation by photosynthetic linear electron flow (NADPH) and by oxidative metabolism of the photosynthates produced (both NADH and NADPH). Indeed, increased carbon availability could also generate an increased carbon flux toward glycolysis and other oxidative pathways, generating reducing power. Nevertheless, the consumption of reducing power by mitochondrial respiration was similar at high or low CO_2_ availability. Still, in the CO_2_ condition, the respiratory cytochrome pathway was specifically decreased in favor of the alternative one through AOX (Figure 3C). The relative increase of the respiratory pathway through AOX compared to the cytochrome pathway is a distinct feature of *C. reinhardtii* compared to other green algae, such as *C. vulgaris* or *C. sorokiniana*, where instead, the opposite was observed (Cecchin et al., 2021). This discrepancy could be explained by the different increase in biomass production, and thus carbon fixation, induced by high CO_2_ availability in *Chlorella* species compared to *C. reinhardtii* when cultivated in the same conditions: ~2.5–2.7 fold increase in biomass accumulation was measured in *C. vulgaris* or *C. sorokiniana* in CO_2_ *vs*. AIR condition. In contrast, only a 69% increase was observed in *C. reinhardtii* (Table S1). The more substantial effect of high CO_2_ availability in *Chlorella* species in carbon fixation might suggest an increased chloroplast demand for NADPH compared to the *C. reinhardtii* case. Differently, in *C. reinhardtii*, the reducing power generated in the mitochondria in excess compared to the requirement for ATP production is dissipated through AOX, being the chloroplast less efficient than *Chlorella* species in consuming reducing power.

In conclusion, high CO_2_ availability positively impacts the productivity of *C. reinhardtii* culture, but only if sufficient light energy is provided. Increased carbon fixation at high CO_2_ availability and high light leads to a decreased protein and starch content per dry weight but increased accumulation of energy‐rich TAGs. The photosynthetic machinery undergoes an adaptation to the increased carbon availability and the increased carbon fixation's demand for ATP and NADPH: PSII light‐harvesting properties, PSI activity, and the chloroplast ATPase content are increased. These adaptations lead to an increased linear electron flow and a switch from the donor to the acceptor side PSI limitation. The decreased consumption of reducing power through the mitochondrial cytochrome respiratory pathway, while the alternative pathway through AOX is increased, suggests that at high CO_2_ availability, ATP synthesis spatially shifts towards chloroplast and cytoplasm. Given the increased availability of photosynthates, glycolysis may represent an essential source for the energetic needs of the cell grown at high light and high CO_2_ availability, but the consequently produced reduced cofactors generated are translocated back to the plastids, rather than being used for mitochondrial respiration, to support carbon assimilation and energy‐rich lipids accumulation.

## AUTHORS CONTRIBUTION

M.B. conceived the work and designed the experimental plan. M.B., S.CA. and Y.LB. supervised experiments. L.Z., M.C., M.P., T.M., G.B., S. CA. and S.CU. performed experiments. Y.LB. and S.CU. performed lipid analysis. M. B. wrote the manuscript with the contribution of L.Z., S.CA. and Y.LB.. All the authors discussed the results, contributed to data interpretation, and commented on and approved the final manuscript.

## FUNDING INFORMATION

This work was supported by the Cariverona Foundation (research grant CARIVERONA‐HABITAT‐2022 n. 2022.0236 to M.B.) and by the ERC Starting Grant SOLENALGAE (679814) to M.B.

## Supporting information


**Table S1.** Comparison of the effect of different CO_2_ availability on biomass accumulation and biomass productivity in *Chlamydomonas reinhardtii*, *Chlorella vulgaris* and *Chlorella sorokiniana*.
Figure S1. Maximum Photosystem II quantum yield at the different light and CO_2_ conditions tested.
Figure S2. Cell concentration at the end of the growth in the different light and CO_2_ conditions tested.
Figure S3. First derivative of the fitted growth curves at the different conditions tested.
Figure S4: Chlorophyll content per cell at the different light and CO_2_ conditions tested.
Figure S5. Western blot analysis of RuBisCO and carbonic anhydrase content per cell.
Figure S6. Western blot analysis of CP43, PsaA, LHCII, and RUBISCO content on a chlorophyll basis in cells grown at 100 or 200 μmol m^−2^ s^−1^.

## Data Availability

The data that support the findings of this study are available from the corresponding author upon reasonable request.
