# Supplementary material for: Interplay between CO2 and light governs carbon partitioning in Chlamydomonas reinhardtii
Source: Physiol Plant. 2024 Nov 19;176(6):e14630. doi: 10.1111/ppl.14630 (PMC11659805; doi:10.1111/ppl.14630)

SUPPLEMENTARY DATA

Table S1. Comparison of the effect of different CO_2 availability on biomass accumulation and biomass productivity in *Chlamydomonas reinhardtii*, *Chlorella vulgaris* and *Chlorella sorokiniana*. The comparison is presented for cells grown in CO2 vs. AIR conditions as described in the main text. * data from *Cecchin, M., Paloschi, M., Busnardo, G., Cazzaniga, S., Cuine, S., Li-Beisson, Y., . . . Ballottari, M. (2021). CO2 supply modulates lipid remodelling, photosynthetic and respiratory activities in Chlorella species. Plant Cell and Environment. doi:10.1111/pce.14074.*

	Fold change biomass accumulation (g l^{-1}) CO2 VS AIR	Fold change biomass accumulation (g l^{-1} day^{-1}) CO2 VS AIR
C. reinhardii	1.69 ± 0.17[a]	2.95 ± 0.30[a]
*C. vulgaris**	2.53± 0.52[b]	4.05 ± 0.90[a,b]
*C. sorokiniana**	2.69± 0.13[b]	3.62 ± 0.24[b]

Figure S1. Maximum Photosystem II quantum yield at the different light and CO_2 conditions tested. ANOVA analysis with post Tuckey post-hoc test ($P < 0.05$) was performed to analyze the data resulting in no significant difference between the different conditions of growth.

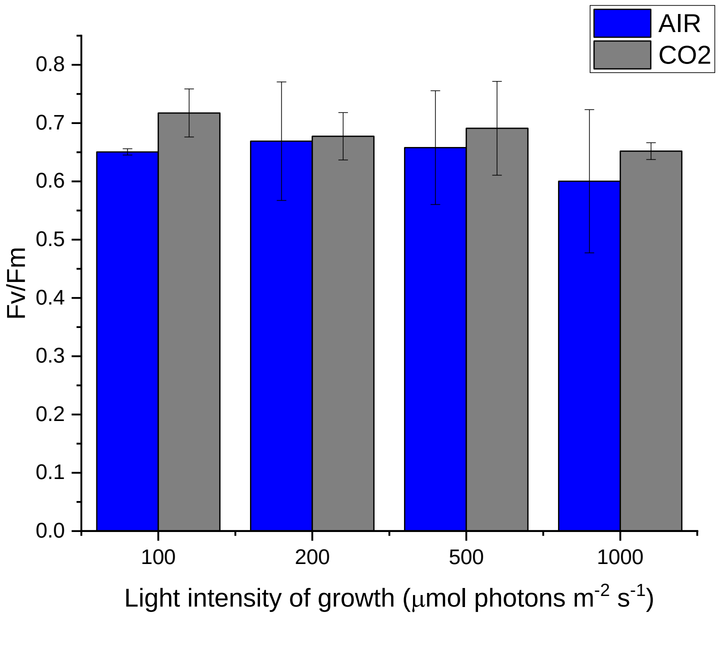

Figure S2. Cell concentration at the end of the growth in the different light and CO2 conditions tested. Significant different values in the different conditions are indicated by different letters according to ANOVA analysis post Tuckey post-hoc test ($P < 0.05$).

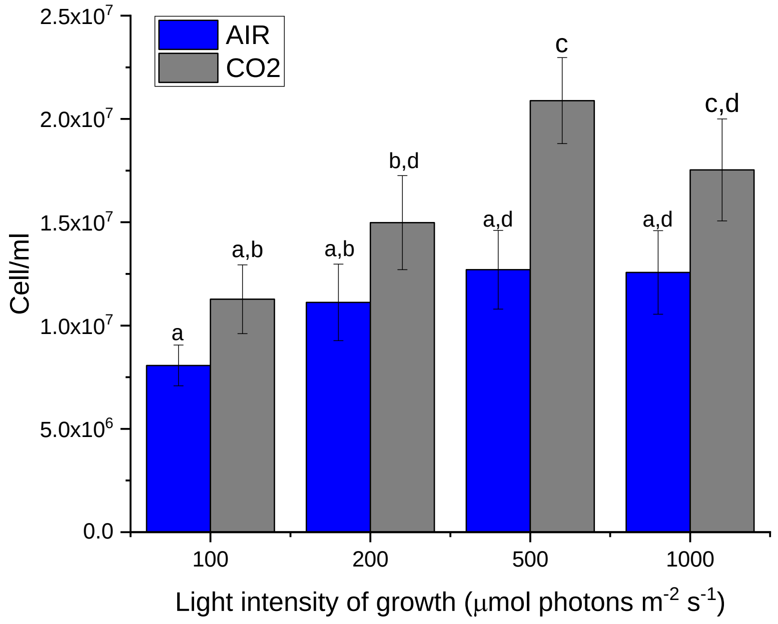

Figure S3. First derivative of the fitted growth curves at the different conditions tested. A) First derivative of the fitted growth curves of the samples grown in AIR conditions. B) First derivative of the fitted growth curves of the samples grown in CO2 conditions.

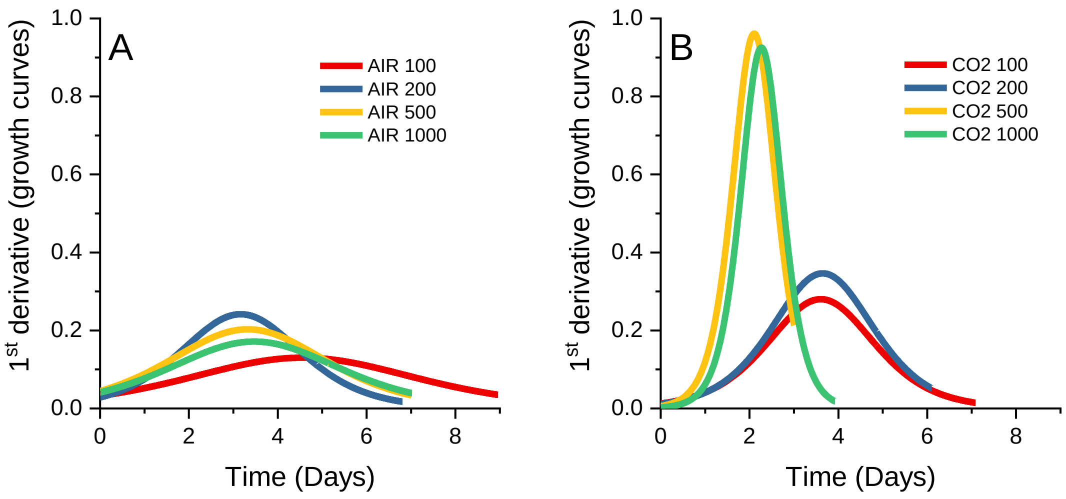

Figure S4: Chlorophyll content per cell at the different light and CO_2 conditions tested. Significant different values in the different conditions are indicated by different letters according to ANOVA analysis post Tuckey post-hoc test ($P < 0.05$).

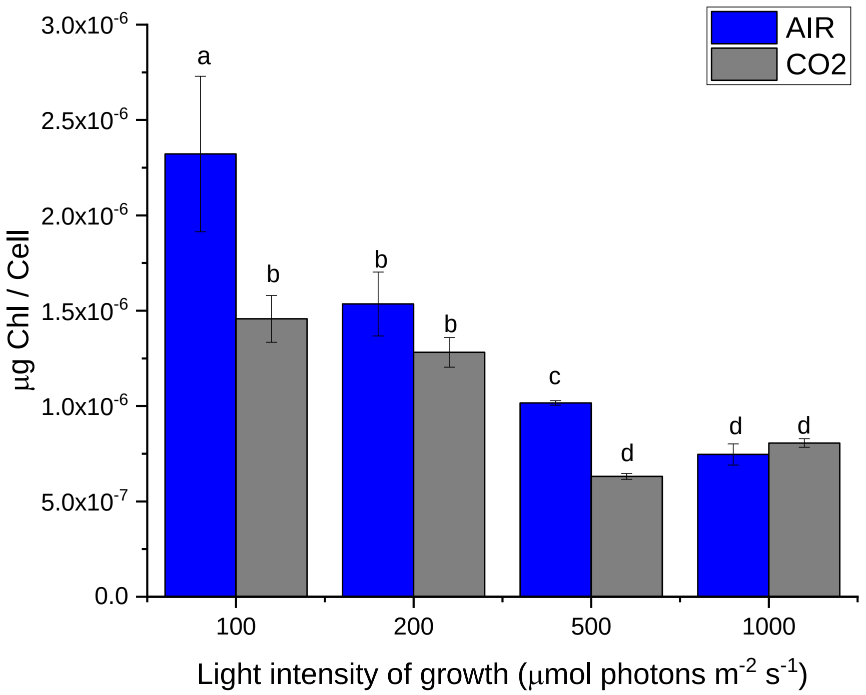

Figure S5. Western blot analysis of RuBisCO and carbonic anhydrase content per cell. RuBisCO and carbonic anhydrase (CAH3) content per cell (A, B) were calculated from densitometric analysis of immunoblotting results reported in Figure 4. Data are means of three biological replicates with standard deviation shown. Significantly different values in CO_2 versus AIR are indicated by ** (P<0.01).

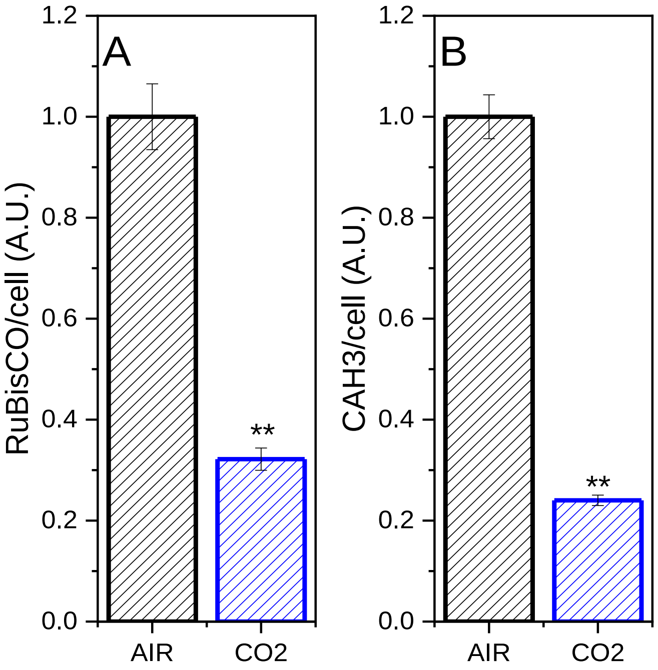

Figure S6. Western blot analysis of CP43, PsaA, LHCII and RUBISCO content on a chlorophyll basis in cells grown at 100 or 200 µmol m^{-2} s^{-1}. A) Immunoblotting results of different proteins involved in the photosynthetic process: Photosystem I subunit PsaA, Photosystem II subunit CP43, LHCII antenna proteins, RuBisCO large subunit (RbcL), B) Densitometric analysis of western blot reported in A) expressed as protein content on a chlorophyll (Chl) basis normalized to AIR condition at 100 µmol m^{-2} s^{-1}. Significantly different values in CO_2 versus AIR are indicated by * ($P<0.05$).

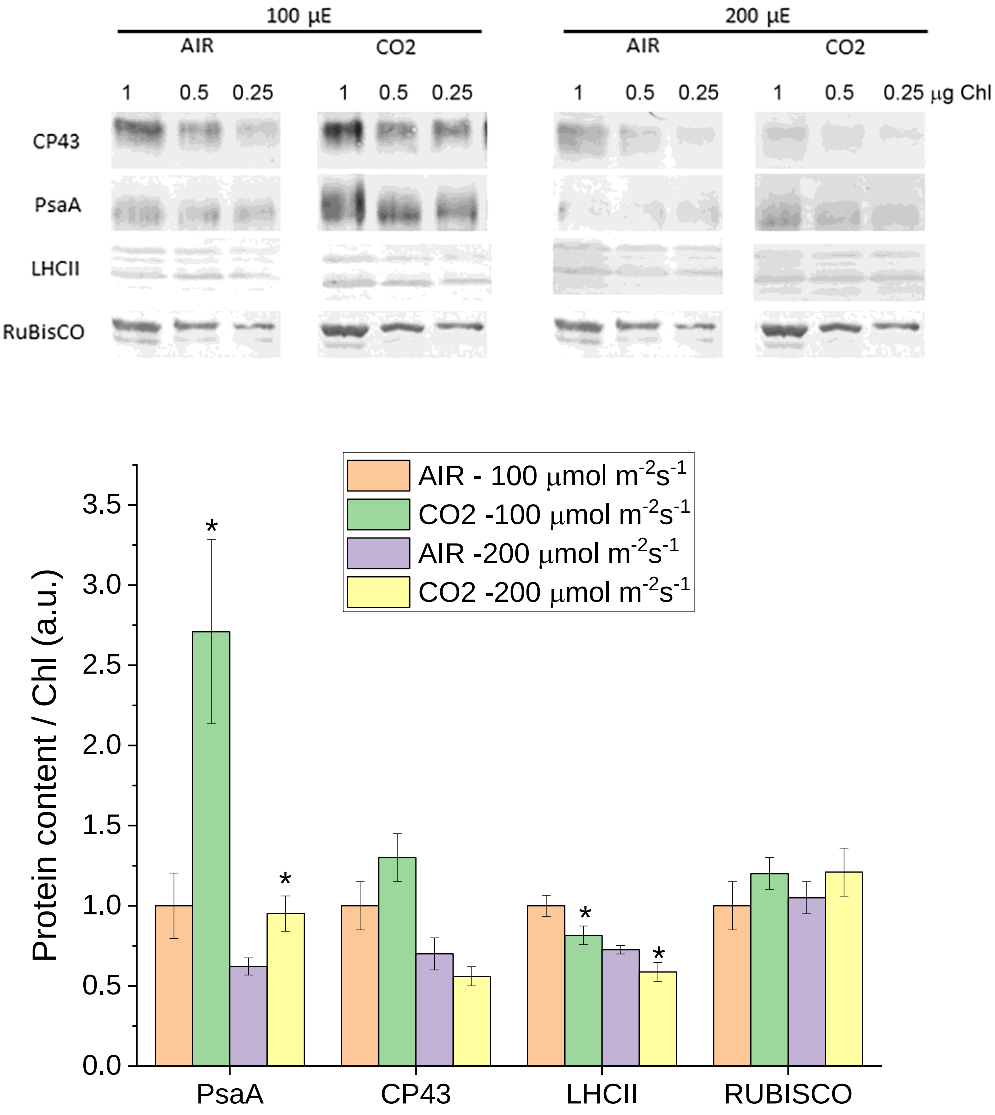

Supplement: Supplementary file 1 — Table S1. Comparison of the effect of different CO2 availability on biomass accumulation and biomass productivity in Chlamydomonas reinhardtii, Chlorella vulgaris and Chlorella sorokiniana. Figure S1. Maximum Photosystem II quantum yield at the different light and CO2 conditions tested. Figure S2. Cell concentration at the end of the growth in the different light and CO2 conditions tested. Figure S3. First derivative of the fitted growth curves at the different conditions tested. Figure S4: Chlorophyll content per cell at the different light and CO2 conditions tested. Figure S5. Western blot analysis of RuBisCO and carbonic anhydrase content per cell. Figure S6. Western blot analysis of CP43, PsaA, LHCII, and RUBISCO content on a chlorophyll basis in cells grown at 100 or 200 μmol m−2 s−1. [file PPL-176-e14630-s001.pdf]
